# Effect of oil drop urine test for polycystic ovary syndrome in Siddha medicine

**DOI:** 10.6026/973206300212314

**Published:** 2025-08-31

**Authors:** Lavanya A., Rathinamala R., Vinodini R., Sathyarajeswaren P., Vinod N.P

**Affiliations:** 1 Research Officer (Siddha) S-II, Siddha Regional Research Institute, Puducherry - 605013, India; 2 Research Officer (Siddha) S-II, ,Siddha Central Research Institute & Hospital, Chennai - 600106; 3 Research Associate (Siddha), Siddha Regional Research Institute, Puducherry - 605013, India; 4 Assistant Director (Siddha) S- IV i/c, Siddha Regional Research Institute, Puducherry - 605013, India; 5 Statistical Assistant, Siddha Central Research Institute & Hospital, Chennai - 600106, India

**Keywords:** Siddha medicine, *Karpa vayu*, Polycystic Ovary Syndrome (PCOS), *Neykkuri*, *Mukkurra iyal*, *Teka ilakkanam*, Body constituents

## Abstract

Polycystic Ovary Syndrome is a heterogeneous disorder with multiple and unclear aetiology and a significant cause of menstrual irregularity, subfertility and infertility. The methodology of diagnosis in Siddha system is based on three humors, Body constituents and eight-fold examinations. Among these parameters, *Neykkuri* (oil drop test), sub test of Urine exmination, a widely used diagnostic and prognostic tool to assess the imbalance of the body's vital humors. This study aims to document the Urine sign - Oil drop test in PCOS and its association with three humours and Body constituents. The results showed a significant association (P=0.016) between three humours and Urine sign which was observed in PCOS patients. This preliminary study in PCOS patients revealed a potential correlation between Urine sign and three humours, highlighting their relevance within Siddha diagnostic principles in the assessment of PCOS.

## Background:

The Siddha system of Medicine is one of the inheritance systems of medicine practiced in South India, especially in Tamil Nadu. It uses a scientific and comprehensive approach to provide preventive, promotive, curative, rejuvenating and rehabilitative healthcare [1]. According to Siddha system, the human body is composed of 96 fundamental principles (*Tattuvam*) and three body humours (*Mukkurram*) is one among them. Three body humors consist of Airy (*Vali*), Fiery (*Alal*) and Watery (*Aiyam*) [2], which contribute to the physical, physiological, and psychological components of an individual. Body constituents (*Teka ilakkanam*) are based on three body humors, forming nine different types of somatotypes. These nine types are brought under 3 major categories, *Vali* constituent, *Alal* constituent and *Aiyam* constituent. An accurate assessment of the body constituents is helpful for evaluating the health status of the patient thus paying attention to diagnosis, treatment and preventive measures. Eight-fold type of clinical assessment - ISMT-4.2.1 (*Envakait tervu*) is the basic diagnostic approach in Siddha system of medicine. The components of Eight-fold type of clinical assessment are Unique Siddha pulse reading method - ISMT-4.2.8 (*Nati*), Examination of Touch/ palpation - ISMT-4.2.5 (*Sparicam*), Examination of Tongue - ISMT-4.2.2 (*Na*), Examination of Colour/ Complexion -ISMT-4.2.6 (*Niram*), *Moli* (Examination of Speech), Examination of Eye - ISMT-4.2.7 (Vili), Examination of Stool (*Malam*), Urine examination - ISMT-4.2.3 (*Muttiram - Nirkkuri*) and Oil Drop Urine Test (*Neykkuri*) [3, 4]. Among these parameters, Oil Drop Urine Test is widely used to assess the imbalance of the body's vital humor (*Vali* (ISMT-1.1.12), *Alal* (ISMT-1.1.23) and *Aiyam* (ISMT-1.1.29)). The oil drop disseminates like a snake in *Vali*, spreads like a ring in *Alal* and stands like a pearl in *Aiyam*. These spread patterns indicate a pathological state of a person [5]. An imbalance of humours is marked by a combined shape such as a ring in a snake, a snake in the ring, a snake and a pearl, or a pearl in the ring. If the oil drop shows all the mentioned characteristics at once or sinks in the urine, it suggests all three humors are affected which indicates the incurable status of a disease [6]. Likewise, many Oil Drop Urine Test patterns describe the diagnosis, severity, and prognosis of the disease condition which are listed in Siddha literature [6]. As an example, the oil drop patterns with Drum, Flag, Pot, Pig, Jungle beast or Potter's wheel shape reflects poor prognosis of disease and sieve shape denotes *Aiyam* diseases that requires immediate treatment. Also, it spreads like the tip of a grass which indicates derangement of *Vali* associated with *Alal*, *etc.* [5, 6].

A pattern of oil spreading is determined by the surface-active molecules and metabolites that are present in the urine sample. The direction of oil spreading may be explained based on paramagnetic molecules that are arranged in a line with the earth's electromagnetic field. The surface-active molecules and other metabolites present in traces are substances that are normally not recordable, but they regulate the spreading pattern of oil. The interfacial tension between the surface-active molecules and viscous forces decides the possibilities of the shape and spreading of oil in urine [7]. Polycystic Ovary Syndrome (PCOS) is a complex disorder characterized by elevated androgen levels, irregular menstruation and/or small cysts on one or both ovaries. The prevalence of PCOS among adolescent females in Tamil Nadu is 18-20% and in India ranges from 3.7- 22.7% [8]. The Rotterdam Criteria which remain the most often used and recognized criteria for the diagnosis of PCOS. Siddha literature describes the clinical symptoms of *Karpa vayu*, which include constipation, low back pain, obesity, missed abortions, irregular menstruation, and dysmenorrhea [9]. The clinical characteristics of PCOS are comparable to the clinical features of *Karpa vayu*. Given the ongoing debate over diagnostic criteria and the gaps in clinical care, there is a pressing need to refine PCOS diagnostic tools. A more precise and individualized approach would improve patient outcomes, ensure timely diagnosis, and provide clearer guidance for both research and treatment. After nearly two decades, revisiting and enhancing the diagnostic framework is essential to better serve patients and advance medical understanding of PCOS. Considering the issue, it is decided to explore a Siddha Oil Drop Urine Test Pattern of diagnosed PCOS patients (Rotterdam Criteria) in a cost-effective manner [10]. This technique is non-invasive, cost-effective, and useful for assessing both diagnosis and prognosis. Therefore, it is of interest to show the effect of oil drop urine test for PCOS in Siddha medicine through this study.

## Materials and Methods:

A cross-sectional analysis was done for 30 PCOS participants who attended OPD of SRRI, as a part of Intra Mural project. This trial was approved by IHEC and registered in Clinical Trials Registry-India (CTRI/2022/03/041489). The standard operative procedures for this analysis are as follows: The physical, mental and functional profile of the study participants was recorded using the standard questionnaire along with socio demographic details. The deranged vital humours of the participants were evaluated with the help of clinical features (Table 1 - see PDF). The clinical features were recorded in the case record form. The patients were advised to have balanced diet and sound sleep before the day of examination. Next day the urine was collected in a sterile 100 ml container. It was poured in 100-150 mm Petri dish under controlled room temperature (25°C ± 2°C) and left without disturbance for 2-3 minute. Then, 1 drop of gingelly oil (≈ 50 µL) was dropped onto the urine surface from a height of 3 cm using a calibrated dropper. The oil dispersion was observed over a 5-minute period without any external disturbances [11].

## Statistical analysis:

Statistical analysis was performed using IBM SPSS software version 26.0. Descriptive statistics in terms of frequency and percentage were used to present the predictions. Chi-square analysis was used to determine the association between different parameters based on 95% confidence interval. The differences were considered as significant if the P value <0.05. True positive (TP) and True negative (TN) predictions were evaluated in study. Oil Drop Urine Test pattern was independently assessed by two assessors and the results were compared with 90% of percentage agreement. The participants who reported a similar pattern in Oil Drop Urine Test correlated with the other findings of measurement were considered True positive (TP), and those with zero relevance to the findings were considered True Negative (TN). TP reflects the successful predictions that help in the part of diagnosis and vice versa [12].

## Results and Discussion:

In the Siddha system of medicine, body constituents, three body humors, and the eightfold assessment are fundamental diagnostic tools used for disease identification, treatment planning, and prognosis. As per Siddhar's philosophy, the three body humors *i.e*, airy, fiery and watery together with the five primordial elements (*Pañcaputam*) form the foundational energies governing human physiology [13]. The predominant humor at the time of embryogenesis determines the individual's body constitution, which remains their physiological baseline throughout life. However, external influences such as climate, diet, lifestyle and stress can disrupt this equilibrium, resulting in pathological changes that manifest through clinical symptoms [14]. According to the theory of humoral pathology in Siddha, disharmony among three body humors is the root cause of disease. These humors influence both physical and mental characteristics, functioning as the link between the macrocosm (universe) and microcosm (individual body). External air corresponds to *Vali*, heat to *Alal*, and water to *Aiyam*. Environmental disruptions especially those affecting *Vali* and *Aiyam* can initiate hormonal disturbances, thereby influencing ovarian and uterine function, which is central to disorders such as PCOS, a condition analogous to PCOS in contemporary medicine. PCOS is a condition involving imbalance in all three humors, predominantly *Vali*, which, when reduced, contributes to symptoms such as sleep apnea, menstrual irregularities, dysmenorrhea, and infertility. *Aiyam* elevation is typically associated with weight gain, sluggish digestion, poor appetite, and disturbed sleep, whereas *Alal* dominance contributes to acne, skin pigmentation, anemia, and emotional disturbances. These patterns closely resemble the spectrum of PCOS symptoms. An exploratory pilot study was conducted at SRRI, Puducherry, to evaluate the safety and efficacy of a classical Siddha formulation (GKM) in managing PCOS in reproductive-aged women. As part of the study procedure, the body constituents, three body humors, and oil drop urine test were documented and analyzed. A cross-sectional assessment was performed to identify correlations among these variables and *Vali*date the Siddha diagnostic approach.

Based on clinical features, *Aiyam* aggravation was seen in 60% of cases. The oil drop urine test reinforced this pattern, showing *Aiyam* dominance in 56.6% of cases, followed by *Alal* (26.6%) and *Alal-Aiyam* (16.7%). A statistically significant association (P = 0.016; Cramer's V = 0.51) was found between the three body humors and the oil drop urine test, indicating a strong relationship. Among the four participants with *Aiyam-Alal* patterns in the urine test, three (75%) also showed corresponding humor patterns clinically. *Aiyam* demonstrated the highest diagnostic agreement, with 92% (12 out of 13 cases) matching between clinical assessment and urine test results. In contrast, only 2 out of 8 *Alal* cases matched (Table 2 (see PDF) & 3 (see PDF)). Interestingly, *Alal-Aiyam*, though not reflected through humor-based assessment, was identified in five participants through the urine test. However, only 13.33% of cases showed association between the body constituent and the oil drop urine test, and no correlation was observed between body constituents and the three body humors, suggesting that constitutional characteristics may not directly reflect current pathological changes in PCOS. This highlights the relevance of dynamic diagnostic tools such as the oil drop urine test and humoral assessment in evaluating disease status more accurately than static constitutional types [15]. Modern diagnostic correlations were also explored. All participants with menstrual irregularities or amenorrhea exhibited decreased *Vali*. Ultrasound confirmed increased ovarian volume and the presence of >12 follicles per ovary, which aligns with Siddha's interpretation of disturbed *Vali* and *Aiyam*. Elevated BMI and lipid profiles reflected *Aiyam* aggravation, while elevated androgen levels (Total Testosterone and Free Androgen Index) were linked to *Alal* dominance, supporting the role of *Alal* in hormonal regulation and metabolic heat production. As documented in the ancient Siddha text *Ñanavettiyan* - 1500, PCOS results from *Aiyam* increase and *Vali* depletion. This pattern was evident in our findings, as most participants exhibited *Alal* dominant body constituent, *Aiyam* predominant humoral imbalance, and *Aiyam* dominant oil drop urine patterns. These findings substantiate Siddha's view of progressive disease transformation and highlight the utility of using combined diagnostic methods for understanding chronic gynecological disorders. Despite these insights, the study had several limitations. Urine samples were collected around 8:00 AM rather than using midstream first-morning urine, as prescribed by Siddha tradition, potentially affecting accuracy. The oil drop test was interpreted by only two observers, introducing potential subjectivity and inter-observer variability. A single urine sample was used per participant, and the study lacked a control group for comparison with healthy individuals. Moreover, the sample size was small (n = 30) and no biochemical or imaging data were cross-referenced, which limits broader clinical *Vali*dation. This integrative approach demonstrates how body constituents, humoral assessment, and the oil drop urine test can offer a comprehensive framework for evaluating *Karpa vayu*, linking classical Siddha knowledge with modern diagnostic perspectives on PCOS.

## Conclusion:

The oil drop urine test (*Neykkuri*) is a non-invasive and cost-effective diagnostic method in Siddha medicine, capable of assessing both the diagnosis and prognosis of diseases without the need for laboratory investigations. This study is the first to document oil drop urine test patterns in PCOS and establish a correlation with PCOS related clinical markers. The results also indicate a strong alignment between traditional Siddha assessments and modern clinical findings. These preliminary results highlight the diagnostic potential of oil drop urine test, warranting further *Vali*dation through larger, multicentric studies with standardized methodologies.
